# Obtaining QM/MM binding free energies in the SAMPL8 drugs of abuse challenge: indirect approaches

**DOI:** 10.1007/s10822-022-00443-8

**Published:** 2022-05-22

**Authors:** Phillip S. Hudson, Félix Aviat, Rubén Meana-Pañeda, Luke Warrensford, Benjamin C. Pollard, Samarjeet Prasad, Michael R. Jones, H. Lee Woodcock, Bernard R. Brooks

**Affiliations:** 1 Laboratory of Computational Biology, National Heart, Lung and Blood Institute, National Institutes of Health, Bethesda, MD 20852 USA; 2 Department of Chemistry, University of South Florida, Tampa, FL 33620 USA

**Keywords:** Host–guest binding, Force-matching, SAMPL8, QM/MM free energy, Binding free energy

## Abstract

Accurately predicting free energy differences is essential in realizing the full potential of rational drug design. Unfortunately, high levels of accuracy often require computationally expensive QM/MM Hamiltonians. Fortuitously, the cost of employing QM/MM approaches in rigorous free energy simulation can be reduced through the use of the so-called “indirect” approach to QM/MM free energies, in which the need for QM/MM simulations is avoided via a QM/MM “correction” at the classical endpoints of interest. Herein, we focus on the computation of QM/MM binding free energies in the context of the SAMPL8 Drugs of Abuse host–guest challenge. Of the 5 QM/MM correction coupled with force-matching submissions, PM6-D3H4/MM ranked submission proved the best overall QM/MM entry, with an RMSE from experimental results of 2.43 kcal/mol (best in ranked submissions), a Pearson’s correlation of 0.78 (second-best in ranked submissions), and a Kendall $\tau$ correlation of 0.52 (best in ranked submissions).

**Supplementary Information:**

The online version contains supplementary material available at 10.1007/s10822-022-00443-8.

## Introduction

The ability to accurately compute free energy is fundamental to most (if not all) of rational drug design [1, 2]. Quantities such as binding affinities, acidity constants, and partition coefficients are intrinsically tied to free energy differences and only highlight this demand. Numerous computational approaches have come to fruition to answer the call for reliable free energy predictions [3–5]. The breadth of rigor encompassed by these techniques within the computational chemistry community is vast, ranging from purely empirical to strictly ab-initio based.

Unfortunately, in practice, the sheer number of ways available to compute free energy differences makes selecting an appropriate method an almost unwieldy task, as test-sets across methodological benchmarks tend to be dissimilar, making “apples-to-apples” comparisons impossible. To address the need for unbiased evaluations of the various free energy methodologies peddled throughout the computational community, the Statistical Assessment of the Modelling of Proteins and Ligands (SAMPL) challenge seeks to systematically appraise the current state of computational approaches for computing free energy [6–11]. One of the most popular components of the SAMPL challenges is the prediction of host–guest binding affinities. The ability to accurately perform a virtual screening for a library of ligands against a potential target protein or properly ranking potential therapeutics against a known binding compound is vital to the drug design process. Thus, it is no surprise that the reliable prediction of binding free energies lies at the heart of rational drug design.

**Fig. 1:**
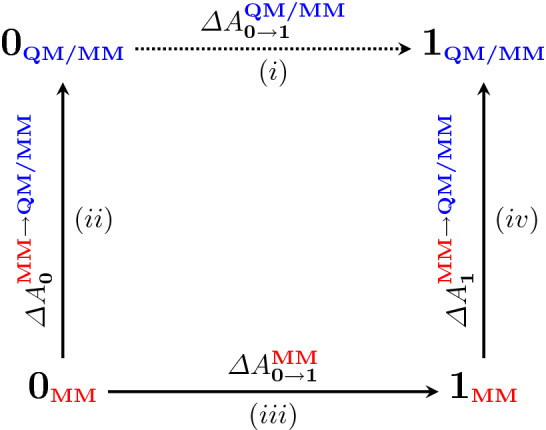
Themodynamic cycle used for computing QM/MM free energy indirectly. By computing each of the legs (*ii*), (*iii*), and (*iv*), it is possible to use the cycle closure to obtain $(iii)+(iv)-(ii)=(i)$, the desired QM/MM free energy difference

Accomplishing this goal requires a faithful and versatile energetic description that encapsulates various chemical moieties and nuanced covalent/non-covalent interactions. Mixed quantum mechanical/molecular mechanical (QM/MM) methods are well-suited for this task, but employing QM/MM Hamiltonians in free energy simulations is often prohibitive, as the cost of performing the relevant dynamics is quite egregious. Even if the computational expense can be ameliorated, the need for alchemical tricks (e.g., soft-core potentials [12, 13]) essentially necessitates the use of MM. Thus, the indirect approach to free energy is a popular strategy for achieving QM/MM free energies by performing the brunt of calculations at a classical level and simply “correcting” to a QM/MM level of theory (see Fig. 1) [14–19]. To keep the requisite free energy differences between levels of theory tenable, it is often desirable to use the Zwanzig equation (e.g., Free Energy Perturbation, FEP, see Eq. 1) [20], as the sampling is all performed classically and the QM/MM energetics can be trivially post-processed. It should be noted that Eq. 1 only works whenever there is sufficient configurational overlap between the MM and QM/MM levels of theory.

1 $$\begin{aligned} \varDelta {A}^{\mathbf{MM }\rightarrow {\mathbf{QM/MM }}}= -\frac{1}{\beta } \log { \left\langle \exp { \left[ -\beta {(U^{\mathbf{QM/MM }} - U^{\mathbf{MM }})} \right] }\right\rangle _{\mathbf{MM }}} \end{aligned}$$

**Fig. 2:**
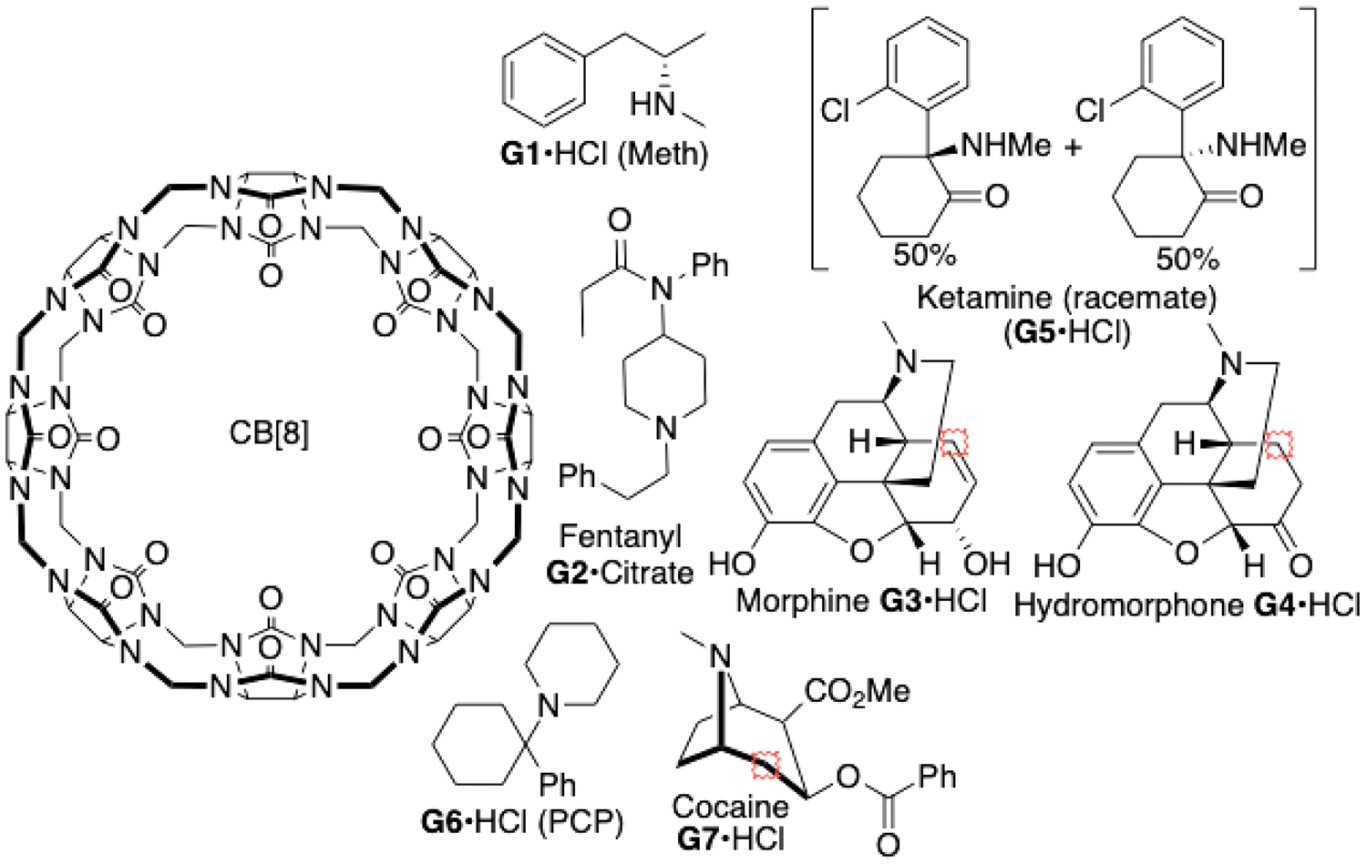
Molecules of interest for the SAMPL8 drugs of abuse CB[8] host–guest binding challenge. Image was provided by the SAMPL coordinators [23, 24]

This work presents a culmination of QM/MM-based attempts at computing binding free energies for the SAMPL8 drugs-of-abuse challenge. This particular challenge involved predicting the binding free energy of several narcotics, such as cocaine, morphine, and fentanyl to curcurbit-[8]-uril [21, 22]. A list of all seven challenge compounds, as well as the host molecule, can be seen in Fig. 2 (see [23]).

Results herein are obtained in a two step process. First, classical parameters are obtained through QM intra-molecular force-matching [25–33], the rationale being that selecting parameters that best reproduce forces at the desired level of QM theory will produce better results through the indirect cycles (i.e., convergence of Eq. 1 will be easier to achieve). Binding free energies are then computed with the force-matched (FM) parameters, and corrections are computed at thermodynamic end states. This manuscript solely focuses on indirectly obtained QM/MM binding free energies. Further discussion on the classical results (e.g., with FM parameters) can be found in a companion work.

## Methods

### Parameterization

All parameters herein are based on the potential energy function utilized by the CHARMM forcefield [34]. Initial host/guest CHARMM parameters were obtained with the Paramchem server [35] and designated herein as the “C36” parameter set. The host parameters were reused from a previous (SAMPL6) competition, designated as “S6” CB[8]. Details of the S6 parameterization process, as well as their overall performance, can be found in [36, 37]. For molecule G5 (Ketamine), we opted to model both the R & S enantiomers of ketamine, which in principal should give identical outcomes when bound to an achiral host in achiral solvent. Since the experimental results were obtained at a pH of 7.4 [38], and the experimental pH of ketamine is about 7.5 [39], both protonated and neutral ketamine were considered, giving a total of 4 parameter sets for G5 (i.e., either R or S enantiomer, and either protonated or neutral). The guest compound p$K_{\text {a}}$values, found in a corresponding literature search, are summarized in SI (Table S1).

As the use of FM potentials proved beneficial in SAMPL6, we opted to incorporate a similar workflow into the present SAMPL8 challenge [37]. That is, all parameters governing bonded degrees of freedom (e.g., bonds, angles, dihedrals, etc.) were fit via QM intramolecular force-matching. Lennard-Jones (LJ) terms were carried over from CGenFF [40] (based on Paramchem assignment), and partial charges were fit via QM charge fitting. The goal of using FM based parameters is that by forsaking transferability, one can create parameters that have higher configurational overlap with the target Hamiltonian of interest (e.g., QM/MM) and thereby allow easier convergence of free energy differences between levels of theory [37].

#### Assigning non-bonded parameters

**Fig. 3:**
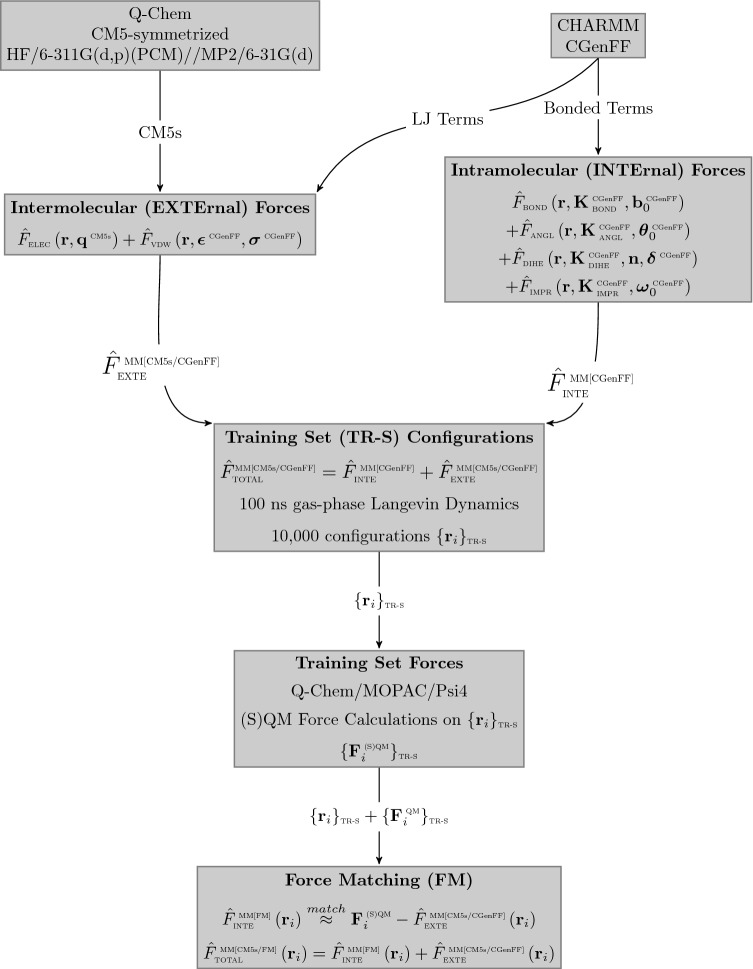
Flowchart used in the intramolecular force-matching process

Using Q-Chem and following along the flowchart shown in Fig. 3, all the guest molecules were subjected to geometry optimizations with MP2/6-31G* [41, 42], whereas the host was optimized with B3LYP/6-31G* [43, 44] to reduce computational expense. We attempted to obtain charges that would be most similar to what would be found in the CGenFF force field, thus a benchmark of 19 small rigid molecules found in CGenFF was performed with various charge fitting schemes and ranked based on RMSE from the original CGenFF charges. The results of the benchmark (details can be found in SI, section S1) demonstrated CM5-symmetrized [45] charges with HF/6-311G** and PCM [46] implicit solvent provided the lowest RMSE from the CGenFF reference, and thus was used in this study. As mentioned prior, LJ parameters were kept consistent with CGenFF via the initial assignment based on the Paramchem server.

#### Assigning bonded parameters

Employing the C36 parameter sets for the guest (and S6 for the host), 100 ns of gas-phase Langevin Dynamics (LD) without non-bonded interaction cutoffs was generated via CHARMM at 300 K with a collision frequency of 5 $\hbox {ps}^{-1}$, a timestep of 1 fs, and a coordinate snapshot saving frequency of 10 ps [34]. From the 10,000 collected configurations per molecule, QM^1^ force calculations were performed on each snapshot at the MP2/6-31G(d) [41] (B3LYP/6-31G(d) for the host), $\omega$B97X-D/def2-SVP, PM6-D3H4, and GFN-2 levels of theory using Psi4 [51], MOPAC [52], and XTB [48] respectively. Classical non-bonded forces (LJ and Coulomb) were removed from the QM-computed force to focus the fit solely on the intramolecular terms (i.e., bonds, angles, dihedrals, etc). Force matching proceeded via the ForceSolve program, which incorporates a Bayesian formalism that obtains parameters by minimizing a log-likelihood function of the observed QM forces [37, 53]. Urey-Bradley terms were omitted from fits, as numerical issues can arise in the force-matching process [54], and the functional form of the potential (e.g., dihedral multiplicities, improper dihedrals where present, etc.) were carried over from the initial Paramchem assignment. In a few cases where force constants for dihedrals were unfeasibly high (e.g., greater than 50 kcal/mol), the multiplicities were tweaked to obtain viable torsional parameters while maintaining reasonably similar residuals. Manual adjustments to dihedrals were based on the FM parameters obtained with PM6-D3H4 and kept consistent across other FM parameter set (i.e., dihedral multiplicities were kept identical during force-matching across different QM/MM Hamiltonian for each molecule). A summary of the various FM parameters can be found in Table 1.

**Table 1:** Breakdown of the different classical Hamiltonians relevant to the QM/MM submissions for the SAMPL8 challenge

Classical FF key	Charge treatment	Bonded treatment
S6	Parameters from SAMPL6 (host only)	
FM(GFN-2)	HF/6-311G**/PCM/CM5s	FM with GFN-2
FM(PM6-D3H4)	HF/6-311G**/PCM/CM5s	FM with PM6-D3H4
FM($\omega$B97X-D)	HF/6-311G**/PCM/CM5s	FM with $\omega$B97X-D/def2-SVP

### Pose generation and system setup

Each of the guest molecules was docked into CB[8] using GalaxyDock [55, 56], with 3–5 unique poses identified per host–guest pairing. Pose refinements were obtained by employing GBMV implicit solvent model in CHARMM with a center-of-mass restraint between the guest and host (vide infra), as well as RMSD restraints on the host and guest individually. Successive short MD simulations followed in which the force constants of the restraints were relaxed.

#### Implicit solvent refinement details

Generated poses were placed into a cylindrical restraint that protruded through the CB8 host, with the *z*-axis of the cylinder defined by the two midpoints of the oxygens along the top and the bottom of the CB[8] host, and a cylinder radius of 6.2 Å. Initial conformations of bound poses were maintained with a heavy-atom RMSD restraint of 200 kcal/mol$\cdot$Å$^{2}$ and with a maximum allowable RMSD of up to 1 Å, whereas an absolute RMSD restraint held the host fixed in Cartesian space about the origin with a force constant of 999 kcal/mol$\cdot$Å$^{2}$. The guest was then further dragged into the host via a flat bottom harmonic restraint between center-of-masses of the host and the guest to keep the COM distance below a specified value (i.e., 2 Å for G3 and G4, 6 Å for G2, and 3 Å for G1, G5, G6, and G7). With an initial force constant of 0.001 kcal/mol$\cdot$Å$^2$, the RMSD restraint on the guest is removed and a series of 5 ps GBMV implicit solvent LD simulations are ran, with the next subsequent simulation increasing the host–guest COM distance force constant by a factor of 10, up to a maximum of 10 kcal/mol$\cdot$Å$^2$ (i.e. five sequential simulations). All of these simulations were run using a 5 $\hbox {ps}^{-1}$ coefficient of friction, without non-bonded cutoffs and a timestep of 1 fs.

#### System setup

Both the bound poses and the individual guests were solvated in a 55 Å cubic water box of TIP3 water molecules. Sodium cations were added as needed to neutralize the system, and 2 additional sodium/chlorine ion pairs were added in order to replicate the experimental ionic strength of 0.15 M. All simulations herein were performed using LD with a timestep of 1 fs, a temperature of 298.15 K, a friction coefficient of 5 $\hbox {ps}^{-1}$, a 1 ps coordinate saving frequency, particle-mesh Ewald for electrostatic treatment, and a polynomial potential switching function between 10 and 12 Å for LJ interactions. Simulations were performed with OpenMM version 7.4.2 on GPUs [57]. No bond or angle constraints were used on either the host or guest. Host–guest bound complexes were maintained via a flat bottom potential with a force constant of 1.5 kcal/mol$\cdot$Å$^2$ with an offset of 5.5 Å to ensure the guest remained bound. Each system underwent a brief 1 ns NPT LD simulation using a Monte-Carlo barostat set to 1 atm with a frequency of 25 steps.

### Free energy simulations

**Fig. 4:**
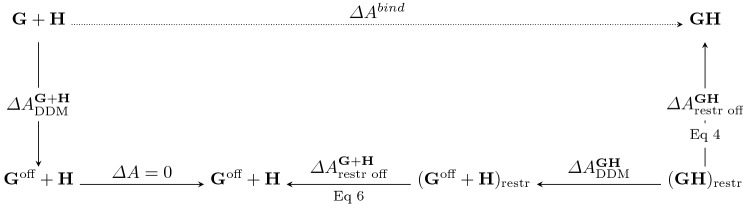
The cycle used for computing the classical binding free energy. The guest bound-state is designated as **GH**, whereas the guest free in solution (or rather, infinitely seperated from the host) is labeled as **G**+**H**. The term “off” is used to indicate the deactivation of non-bonded (e.g., electrostatic and van der Waals) interactions on the guest compound. Subscripts ”restr” denotes a restraining potential is active, and “restr off” refers to the deactivation of the restraining potential . See Eq. 2 for a summary equation

All classical binding free energies were obtained through the scheme outlined in Fig. 4.Starting from the guest–host complex (**GH**, top right, Fig. 4), the guest ${\mathbf {G}}$ is bound within the host ${\mathbf {H}}$ (generation of binding poses is described in the next section). To ensure that the guest remains within the host, a flat-bottom harmonic COM restraint is applied between the guest and the host, corresponding to a free energy contribution $\varDelta A^{{{\mathbf {G}}}{{\mathbf {H}}}}_\text {restr off}$ (Eq. 4). The so-called “double-decoupling” method (DDM) [58, 59], in which electrostatics and van der Waals interactions are sequentially disabled on the guest compound, is used to make the guest “vanish” from inside the host. Then, the free energy of deactivating the COM restraint for the unbound guest is accounted for via the free energy of changing to a standard state $\varDelta A^{{\mathbf {G}}+{\mathbf {H}}}_\text {restr off}$ (Eq. 6) [36]. Since the guest has no intermolecular interactions, the environment is switched from guest–host complex to guest free in aqueous solution without incurring any energetic penalty. At this point, the guest has intermolecular interactions restored through a second DDM calculation^2^.

2 $$\begin{aligned} \varDelta {A}^{\text {bind}}&= \varDelta {A}^{\mathbf {G+H}}_{\text {DDM}} -\varDelta {A}^{\mathbf {G+H}}_{\text {restr off}} - \varDelta {A}^{{{\mathbf {G}}}{{\mathbf {H}}}}_{\text {DDM}} + \varDelta {A}^{{{\mathbf {G}}}{{\mathbf {H}}}}_{\text {restr off}} \end{aligned}$$

Using the DDM approach, electrostatics on the guest molecules were gradually “shut-off” over 5 simulations ($\lambda$-states 0–5), followed by the deactivation of guest van der Waals interactions over an additional 10 simulations ($\lambda$-states 5–15). Details of the 16 successive $\lambda$-states are listed in Table 2. Deactivation of the guest electrostatics was accomplished by simply scaling partial charges on the guest solute, whereas van der Waals deactivation was performed with the so called “soft-core” LJ potential as given by the following equation,

3 $$\begin{aligned}&U_{\lambda _{\mathrm {vdw}}}^{\mathrm {LJ}} (r_{ij}) = 4 \lambda _\text {vdw}\varepsilon _{ij} \left( \left( \frac{\sigma }{r_{ij} + \sigma _{ij}(1-\lambda _{\mathrm {vdw}})} \right) ^{12} \right. \nonumber \\&\quad \left. - \left( \frac{\sigma }{r_{ij} + \sigma _{ij}(1-\lambda _{\mathrm {vdw}})} \right) ^{6} \right) . \end{aligned}$$

All 16 alchemical simulations were performed independently and simultaneously. Alchemical functionality was provided through a specially modified variant of the Alchemy module in OpenMM Tools based on fixes provided in one of the last SAMPL challenges [60].

**Table 2:** $\lambda$-state schedule, with accompanying decoupling parameters for van der Waals ($\lambda _\mathrm {vdW}$) and electrostatic ($\lambda _\mathrm {elec}$) interactions defining each $\lambda$-state

$\lambda$-states	0	1	2	3	4	5	6	7	8	9	10	11	12	13	14	15
$\lambda _{\mathrm {vdW}}$	1.0	1.0	1.0	1.0	1.0	1.0	0.81	0.64	0.49	0.36	0.25	0.16	0.09	0.04	0.01	0.00
$\lambda _{\mathrm {elec}}$	1.0	0.8	0.6	0.4	0.2	0.0	0.0	0.0	0.0	0.0	0.0	0.0	0.0	0.0	0.0	0.0

Starting from snapshots obtained from the final step of the aforementioned NPT equilibration, each lambda-state underwent a 500 steps minimization. An NVT equilibration run of 1 ns followed, based on the consensus box-length obtained from the second half of the NPT equilibration. From this, production simulations were run for 15 ns, giving a total of 15,000 snapshots per lambda-state. Energies for each lambda-state were calculated on the fly per snapshot using the AlchemyFlow module from the RickFlow in-house code [61], and the deactivation free energies $\varDelta {A}_{\text {DDM}}$ were computed via MBAR [62].

#### Restraint free energy

To account for the effect of the flat-bottom potential on the binding free energy, we consider the two contributions to the free energy: (1) $\varDelta {A}^\mathbf{GH }_{\text {restr off}}$, the free energy of turning the restraint off at $\lambda =0$ and (2) $\varDelta {A}^{\mathbf{G }+\mathbf{H }}_{\text {restr off}}$, the free energy of turning off the restraint at $\lambda =15$.

For $\lambda =0$, host–guest interactions are unperturbed, and for a well-bound system the COM distance between the guest and host stays fairly stable. In this situation, the free energy of turning off the restraint can be determined using the FEP method, which yields

4 $$\begin{aligned} \varDelta {A}^\mathbf{GH }_{\text {restr off}}=-\frac{1}{\beta } \log {\left<\exp \left[ -\beta (\varDelta {U}_{\text {restr}}^{\text {on}\rightarrow \text {off}})\right] \right>_{\lambda =0}} \end{aligned}$$

where

5 $$\varDelta {U}_{\text {restr}}^{\text {on}\rightarrow {\text {off}}}(r) = \left\{ \begin{array}{ll} 0 \quad & {\text {if}} \quad r < 5.5 \, \text{\AA} \\ -\frac{1}{2}K(r-5.5)^2 \quad & {\text {if}} \quad r \ge 5.5 \, \text{\AA} \end{array} \right.$$

At $\lambda =15$, the guest electrostatics and van der Waals terms are deactivated (i.e., $\lambda _{\text {vdw}}=\lambda _{\text {elec}} = 0$). Therefore, the flat-bottom restraint will only act as a restriction of the space the guest explores. Hence, the free energy of deactivating the COM restraint $\varDelta {A}^{\mathbf{G }+\mathbf{H }}_{\text {restr off}}$ corresponds to the free energy of changing to standard state (e.g., changing from the effective volume explored through the COM restraint, $V_{\text {eff}}$, to the concentration corresponding to the standard state, $V_{0}$). Based on the work of [63], we can compute the free energy of releasing the restraint as

$$\begin{aligned} \varDelta {A}^{\mathbf{G }+\mathbf{H }}_{\text {restr off}}=-\frac{1}{\beta }\log {\frac{V_{0}}{V_\text {eff}}}. \end{aligned}$$

where $V_0=1661$ $\hbox {AA}^3$ as the volume corresponding to standard state concentration. Using this, and approximating (albeit, an overestimation) $V_{\text {eff}}$ as

$$\begin{aligned} V_\text {eff}&=\int _{{\mathbb {R}}^3} e^{-\beta {U_\text {restr}(\mathbf {{\mathbf {r}}})}}d\mathbf {{\mathbf {r}}} \\&\approx \frac{4}{3}\pi \left( r_\text {max}^3 - r_\text {min}^3\right) , \end{aligned}$$

we arrive at

6 $$\begin{aligned} \varDelta {A}^{\mathbf{G }+\mathbf{H }}_{\text {restr off}} =-\frac{1}{\beta }\log {\left( \frac{V_0}{\frac{4}{3}\pi (r_{\text {max}}^{3}-r_{\text {min}}^{3})}\right) } \end{aligned}$$

where $r_{\text {max}}$ is the maximum observed COM distance between the host and guest, and $r_{\text {min}}$ the minimum observed COM distance between host and guest [59, 63–65]. Values for $r_\text {min}$ and $r_\text {max}$ are found in SI Table S5, with the largest $r_\text {min}$ at 0.27 Å, and thus having a maximum contribution of about 0.08 Å^3^.

#### G5 p$K_{\text {a}}$ correction

**Fig. 5:**
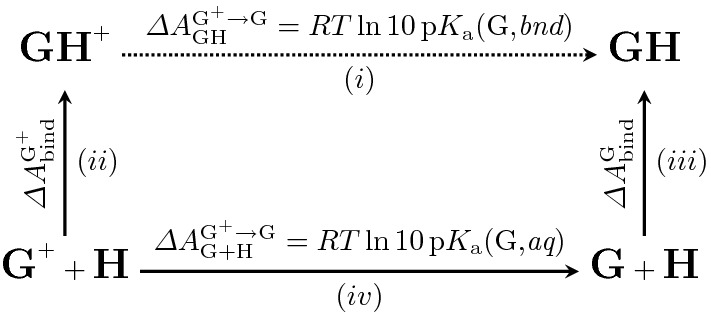
Thermodynamic cycle used to indirectly compute the bound ligand p$K_{\text {a}}$. Quantities (*ii*) and (*iii*) are binding free energies, computed as described in section ‘Free energy simulations’, and (*iv*) is known from the guest p$K_{\text {a}}$

As mentioned prior, both protonated and neutral ketamine (G5) were considered as to incorporate relevant p$K_{\text {a}}$ effects on the binding free energy. Given that the ketamine p$K_{\text {a}}$ in solution is known (p$K_{\text {a}}$(aq) = 7.5, which is close to the experimental pH of 7.4), the p$K_{\text {a}}$ of bound-state ketamine can be indirectly obtained through computing both the protonated and neutral binding free energies ($\varDelta {A}^{G^+}_{bind}$ and $\varDelta {A}^{G}_{bind}$, respectively), and applying the thermodynamic cycle seen in Fig. 5.

Simple manipulation allows us to find $\varDelta {A}^{G^+\rightarrow G}_{GH} = (iv)+(iii)-(ii)$, and therefore we arrive at the following expression for the bound state p$K_{\text {a}}$:

7 $$\begin{aligned} \text {p}K_{\text {a}}(\text {G,}{} \textit{bnd})= \text {p}K_{\text {a}}(\text {G,}{} \textit{aq}) +\frac{\varDelta \varDelta {A}_{\text {bind}}^{G^+\rightarrow G}}{RT\ln {10}}. \end{aligned}$$

Finally, the pH dependent binding free energy is determined by the equation [66, 67],

8 $$\begin{aligned} \varDelta {A}^{\text {pH}=7.4}_{\text {bind}} =&\varDelta {A}_{\text {bind}}^{\text {G}^+} - \frac{1}{\beta }\log {\left[ \frac{1+10^{7.4-\text {p}K_{\text {a}}(\text {G,}{} \textit{bnd})}}{1+10^{7.4-\text {p}K_{\text {a}}(\text {G,}{} \textit{aq})}}\right] } \end{aligned}$$

9 $$\begin{aligned} =&\varDelta {A}_{\text {bind}}^{\text {G}} - \frac{1}{\beta }\log {\left[ \frac{1+10^{\text {p}K_{\text {a}}(\text {G,}{} \textit{bnd})-7.4}}{1+10^{\text {p}K_{\text {a}}(\text {G,}{} \textit{aq})-7.4}}\right] } \end{aligned}$$

### Indirect QM/MM practical scheme

**Fig. 6:**
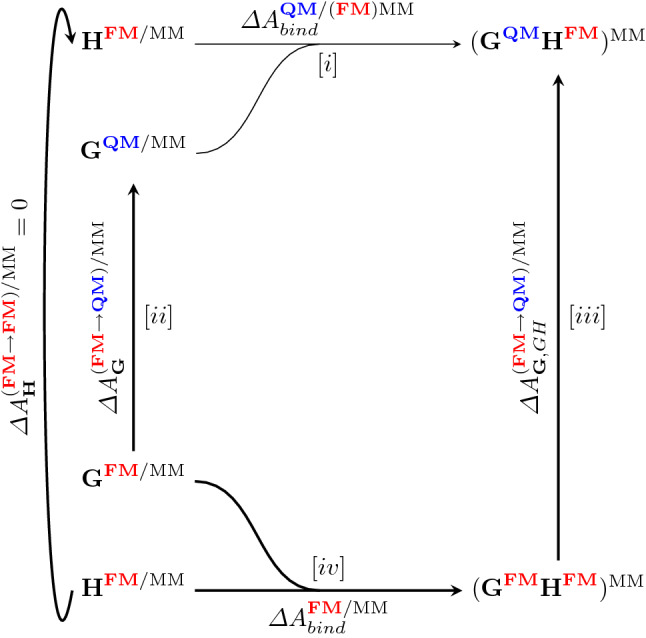
The cycle used for the indirect calculation of QM/MM binding free energies. **H** and **G** respectively designate the host and guest systems, and (**G** **H**) the guest–host complex

After calculating the classical binding free energies with FM potentials, we then aimed to compute QM/MM binding free energies. The cycle of Fig. 1 can be extended for obtaining QM/MM binding free energies as shown in Fig. 6.

In this case, we chose to only perform the QM/MM correction on the guest compounds. Although there is no conceptual barrier for correcting the host as well, the size of the host would make the convergence of these QM/MM free energy corrections dubious at best. The contributions required to obtain the indirect QM/MM binding free energy are summarized in the following:

10 $$\begin{aligned}&\varDelta {A}^{\mathbf{QM/MM }/(\mathbf{MM })MM}_{\text {bind}} = \varDelta {A}^{\mathbf{MM }/MM}_{\text {bind}}\nonumber \\&\quad +\varDelta {A}^{(\mathbf{MM }\rightarrow \mathbf{QM/MM })/MM}_{\mathbf{G },\text {GH}} -\varDelta {A}^{(\mathbf{MM }\rightarrow \mathbf{QM/MM })/MM}_{\mathbf{G }} \end{aligned}$$

Calculating the free energy difference between the MM and QM/MM levels of theory is done through FEP (Eq. 1). To this end, 10 ns of NVT LD simulation were performed for every guest both in bound state and free in solution, saving out a total of 10,000 configurational snapshots per end-state per guest. Four QM/MM methods were selected as target levels of theory, consisting of two SQM (PM6-D3H4 and GFN-2) and two DFT ($\omega$B97X-D and BLYP) approaches. For the DFT based methods, the MM regions were treated as either a field of point charges or by “smearing” charges through fitting to delocalized Gaussians (i.e., Gaussian blurring) [68]. A blur width of 1 Å was used for all QM/MM calculations with Gaussian blurring. A summary of the various levels of theory, MM charge treatments, and software used for computation can be found in Table 3. Standard deviations of the free energy difference were computed via block averaging using 10 blocks of 1000 snapshots each.

**Table 3:** List of target QM/MM levels of theory and the MM levels of theory through which the corrections are computed

Key	QM level of theory	MM treatment	Host treatment	Corrected from	Software
PM6-D3H4	PM6-D3H4	Electrostatic potential	S6	FM(PM6-D3H4)	MOPAC16 [52]
GFN-2	GFN-2	Point charges	S6	FM(GFN-2)	XTB [48]
$\omega$B97X-D	$\omega$B97X-D/def2-SVP	Point charges	S6	FM($\omega$B97X-D)	Q-Chem 5.2 [69]
$\omega$B97X-D[blur]	$\omega$B97X-D/def2-SVP	Gaussian blur	S6	FM($\omega$B97X-D)	Q-Chem 5.2
BLYP	BLYP/6-31G*	Point charges	S6	FM($\omega$B97X-D)	Q-Chem 5.2
BLYP[blur]	BLYP/6-31G*	Gaussian blur	S6	FM($\omega$B97X-D)	Q-Chem 5.2

Since QM/MM Ewald is not fully supported for all the QM levels of theory employed, QM/MM energies were evaluated without cutoffs or Ewald. Instead, following the logic of Refs. [37] and [70], classical energies were also obtained with and without cutoffs or Ewald, and the difference of the two was used to obtain the so-called “PBC” energy ($U^{\text {MM}}_{\text {PBC}}=U^{\text {MM}}_{\text {w/PBC}} - U^{\text {MM}}_{\text {no\,PBC}}$). From this, the relevant energies for evaluating the Eq. 1 were setup as $U^{\text {MM}}=U^{\text {MM}}_{\text {no\,PBC}}+U^{\text {MM}}_{\text {PBC}}$ and $U^{\text {QM/MM}}=U^{\text {QM/MM}}_{\text {no\,PBC}} + U^{\text {MM}}_{\text {PBC}}$, giving $\varDelta {U}^{\text {MM}\rightarrow \text {QM/MM}}=\varDelta {U}^{\text {MM}\rightarrow \text {QM/MM}}_{\text {no\,PBC}}$. Inherently, the underlying assumption is that the contributions from Ewald for QM/MM and MM do not differ drastically. In the case of the two SQM/MM calculations, the energy of the MM environment as well as the QM/MM van der Waals interactions between solute-environment (which are modelled with classical van der Waals potentials) were added in post-processing through CHARMM.

### QM/MM correction metrics

Although a plethora of methods exist for evaluating the convergence quality of a free energy calculation, most require sampling both end states of interest. Since sampling the QM/MM surface is prohibitive, we restrict our focus to metrics applicable to “one-sided” (i.e., only requiring sampling of the FM/MM configurational space) approaches. In previous work, it has been shown that the standard deviation of potential energy difference between FM/MM and QM/MM, as well as the so-called bias measure, can give insight as to when free energy calculations with FEP fail. In regards to $\sigma {\left[ \varDelta {U}^{(\text {FM}\rightarrow \text {QM)/MM}}_{\text {FM/MM}}\right] }$ (i.e., the standard deviation of the potential energy difference from the FM/MM ensemble), larger values are associated with poor convergence due to a sparse collection of snapshots truly contributing to the exponential average (i.e., snapshots occupying the lower energy tail of the $\varDelta {U}$ distribution). As a rule of thumb, keeping $\sigma {\left[ \varDelta {U}^{(\text {FM}\rightarrow \text {QM)/MM}}_{\text {FM/MM}}\right] }\le 4k_{B}T$ ($\approx 2.4$ kcal/mol) is desirable (the smaller the better).

The second metric, the bias measure, indicates the presence of bias in a free energy estimate [71–73]. By assuming that the spread of $\varDelta {U}^{(\text {FM}\rightarrow \text {QM)/MM}}$ is similar between FM/MM and QM/MM surfaces, the bias measure can be applied to our one sided approach, taking the form

11 $$\begin{aligned} \varPi&=\sqrt{{\mathbf {W}}_{L}\left[ \frac{1}{2\pi }\left( N-1\right) ^2\right] }\nonumber \\&\quad - \sqrt{2\beta \left( \left<\varDelta {U}^{\left( \text {FM}\rightarrow \text {QM}\right) /\text {MM}}\right>_{\text {FM/MM}}-\varDelta {A}^{\left( \text {FM}\rightarrow \text {QM}\right) /\text {MM}}\right) } \end{aligned}$$

where ${\mathbf {W}}_L$ is the Lambert function, and $\varDelta {A}^{\left( \text {FM}\rightarrow \text {QM}\right) \text {/MM}}$ is the FEP estimate evaluated with a configurational sampling of size N. Following the recommendations of Refs. [72, 73], $\varPi$ should be greater than 0.5 (the larger the better). Take note that this condition is necessary but not sufficient: although failing to have a $\varPi >0.5$ or a $\sigma {\left[ \varDelta {U}^{(\text {FM}\rightarrow \text {QM)/MM}}_{\text {FM/MM}}\right] }\le 4k_{B}T$ can cast doubt onto the converge of the FEP estimate, satisfying both criteria does not guarantee convergence.

## Results and discussion

### Overview of results

**Fig. 7:**
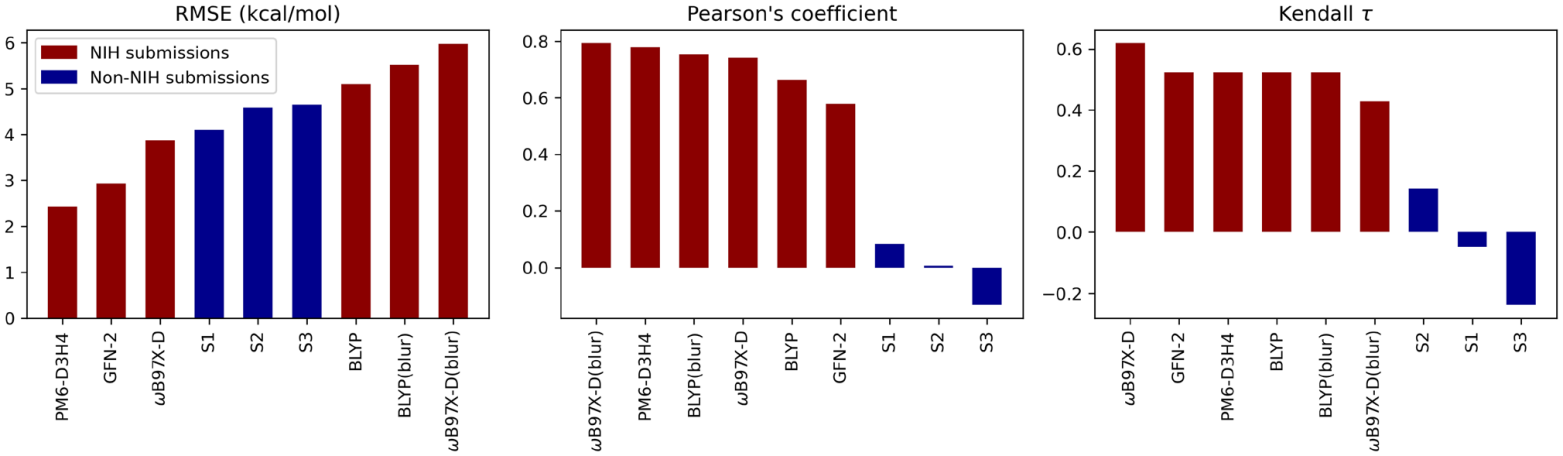
Statistical measurements ranked for all QM/MM submissions. Non-NIH method’s names were given as follows in the SAMPL Github (see [24]). S1: GFN2-xTB/MetaMD/GBSA/ensemble/Nobuffer, S2: GAFF-RESP/TIP3P/MD/xtb-GFN2B/Boltz-Avg, S3: GAFF-RESP/TIP3P/MD-Classical/xtb-GFN2B

Results for the 6 QM/MM submissions, as well as the three FM-based submissions they are corrected from, are found in Table 4 and Fig. 7. From a cursory glance, a few observations can be made. First, the PM6-D3H4 submission is easy to discern as our best QM/MM submission (as well as our best submission overall), with a RMSE of 2.4 kcal/mol. Similar success was achieved using PM6-D3H4 in the SAMPL6 challenge by the Ryde Group [74], and served as inspiration for utilitizing it in SAMPL8. Predictions based on GFN-2 also performed rather well^3^ with a RMSE of 2.9 kcal/mol. Although correlation statistics are comparatively high amongst the QM/MM submissions ($\rho$ is between 0.58–0.79 [75] and Kendall-$\tau$ is between 0.43–0.62 [76]), RMSE is the more indicative metric of success due to the rather small sample size of 7.

**Table 4:** Binding free energies obtained for each guest molecule, in kcal/mol, and statistical quantities for each methods

Method	G1	G2	G3	G4	G5	G6	G7	RMSE	Pearson	Kendall	ME
FM(PM6-D3H4)	− 12.0 ± 0.6	− 13.5 ± 1.2	− 16.4 ± 1.1	− 14.2 ± 0.5	− 12.5 ± 0.6	− 18.4 ± 1.3	− 9.8 ± 1.6	3.61	0.81	0.62	− 3.26
FM(GFN-2)	− 13.7 ± 0.7	− 13.9 ± 0.9	− 16.5 ± 0.7	− 15.5 ± 0.9	− 11.3 ± 0.7	− 20.0 ± 0.7	− 14.7 ± 0.9	5.16	0.48	0.43	− 4.52
FM($\omega$B97X-D)	− 12.4 ± 1.0	− 10.4 ± 0.4	− 16.1 ± 0.5	− 15.3 ± 0.7	− 10.9 ± 1.9	− 18.1 ± 2.1	− 11.2 ± 0.8	3.69	0.62	0.33	− 2.92
PM6-D3H4^‡^	− 9.4 ± 1.1	− 11.8 ± 2.5	− 12.2 ± 2.1	− 11.2 ± 2.2	− 9.7 ± 3.5	− 18.7 ± 1.7	− 5.8 ± 2.7	2.43	0.78	0.52	− 0.69
GFN-2*	− 7.9 ± 0.8	− 7.4 ± 1.0	− 9.4 ± 0.9	− 11.4 ± 1.1	− 18.7 ± 3.6	− 12.6 ± 0.8	− 10.1 ± 1.5	2.94	0.58	0.52	− 0.50
$\omega$B97X-D	− 9.1 ± 0.5	− 12.6 ± 1.7	− 15.5 ± 0.8	− 9.7 ± 1.0	− 10.2 ± 1.8	− 18.1 ± 1.0	− 0.4 ± 2.1	3.86	0.74	0.62	− 0.23
$\omega$B97X-D(blur)	− 11.0 ± 0.5	− 15.0 ± 1.8	− 19.0 ± 0.9	− 20.8 ± 0.9	− 14.6 ± 4.0	− 20.6 ± 1.0	− 3.9 ± 2.0	5.98	0.79	0.43	− 4.42
BLYP	− 9.9 ± 0.5	− 11.9 ± 1.6	− 15.7 ± 0.8	− 14.5 ± 0.9	− 8.7 ± 2.6	− 17.3 ± 1.0	3.1 ± 2.2	5.09	0.66	0.52	− 0.13
BLYP(blur)	− 11.6 ± 0.5	− 13.0 ± 1.6	− 17.8 ± 1.0	− 21.1 ± 1.0	− 14.5 ± 0.8	− 19.4 ± 1.0	− 4.0 ± 2.3	5.52	0.75	0.52	− 3.02
Exp.	− 7.05	− 9.94	− 11.6	− 11.2	− 12.3	− 14.1	− 7.79	–	–	–	–

Method names starting with “FM” represent results obtained prior to correction. Detailed summary for each method can be found in Tables 1 and 3

^‡^Ranked submission

Some noteworthy observations can be made about the effects of correcting FM to QM/MM on our SAMPL8 submissions. First, FM(PM6-D3H4) did reasonably well, but improved remarkably after the QM/MM correction step. This is not the first time that PM6-D3H4 has shown good results for binding free energies in the SAMPL challenges [36]. The semi-empirical GFN-2 was a popular QM/MM choice in this challenge, with 3 other GFN-2 based methods submitted by other groups. However, our GFN-2 results outperformed these in every single metric of interest (see Table 5).

**Table 5:** Table showing the change in the correction metrics $\varPi$ and $\sigma$ for computing $\varDelta {A}^{\text {MM}\rightarrow \text {QM}}$ in gas-phase, with $\varDelta \varPi =\varPi ^{\text {FM/MM}}-\varPi ^{\text {C36/MM}}$ and $\varDelta \sigma =\sigma ^{\text {FM/MM}}-\sigma ^{\text {C36/MM}}$

Mol ID	PM6-D3H4		GFN-2		BLYP/6-31G*		$\omega$B97X-D/def2-SVP	
	$\varDelta {\varPi }$	$\varDelta {\sigma }$	$\varDelta {\varPi }$	$\varDelta {\sigma }$	$\varDelta {\varPi }$	$\varDelta {\sigma }$	$\varDelta {\varPi }$	$\varDelta {\sigma }$
G1	0.98	− 0.59	1.33	− 0.94	1.16	− 0.97	1.47	− 1.21
G2	1.15	− 0.59	1.56	− 0.99	0.65	− 0.66	1.49	− 1.43
G3	1.44	− 1.44	2.86	− 1.89	1.60	− 1.10	2.77	− 2.47
G4	1.13	− 0.76	1.02	− 0.76	0.43	0.16	2.13	− 1.53
*R*-G5$^\circ$	0.24	− 0.03	1.34	− 0.21	− 1.75	4.89	0.04	5.07
*S*-G5$^\circ$	− 0.51	0.82	0.56	− 0.19	0.23	0.33	0.74	− 0.80
*R*-G5$^+$	− 1.80	− 0.91	0.73	− 0.90	0.21	0.80	1.43	0.05
*S*-G5$^+$	0.20	− 0.53	1.43	− 0.94	0.00	0.11	0.94	− 0.51
G6	0.82	− 0.70	1.40	− 1.02	1.55	− 0.78	1.69	− 1.15
G7	− 0.65	1.46	− 0.82	1.48	− 0.24	0.54	− 0.33	0.17

The FM(GFN-2) results were somewhat poor, but drastically improved upon correction (RMSE went from 5.16 to 2.94 kcal/mol). The results for correcting FM to QM/MM with DFT, however, seemed to either offer little improvement (e.g., going from FM($\omega$B97X-D) to $\omega$B97X-D/def2-SVP), or worsen results. Of particular note is the systematically poor performance of DFT in predicting the G7 binding free energy. In previous work, we found underestimates of this magnitude, to the point of suggesting non-binding, were indicative of a problem with the FM parameters (vide infra section ‘Metrics for the indirect approach’).

**Fig. 8:**
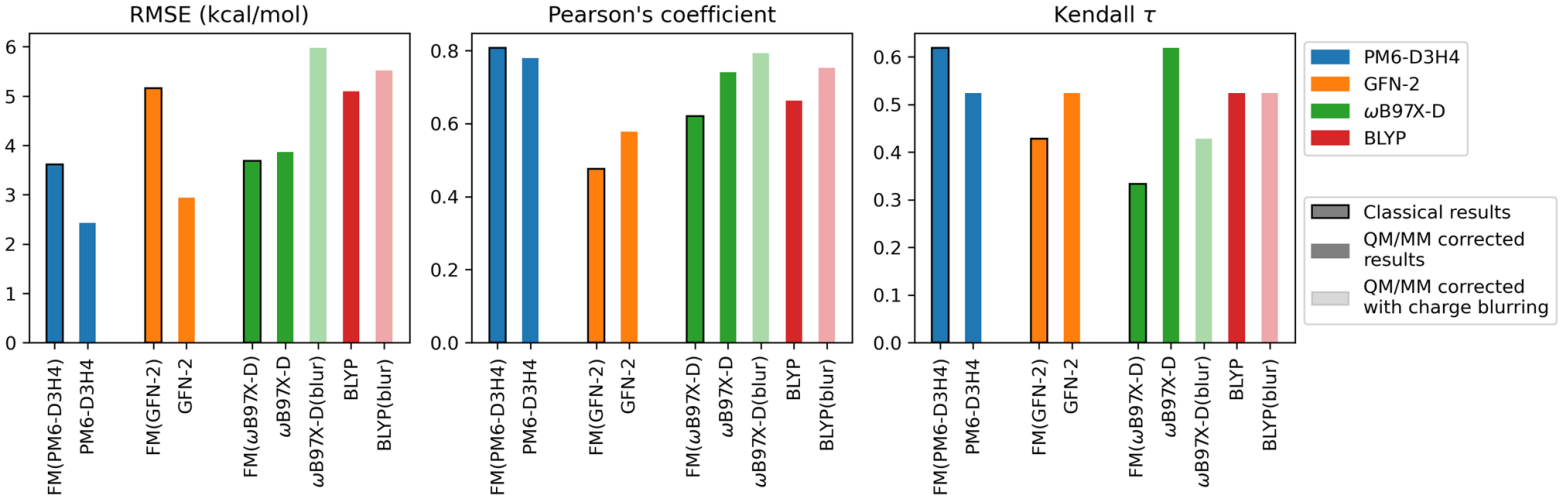
Bar graphs showing the relevant statistics for each QM/MM submission, as well as the associated classical (FM) submission for which QM/MM corrections were added. Results for the FM submissions are outlined in black, and the QM/MM blurred results are translucent

**Fig. 9:**
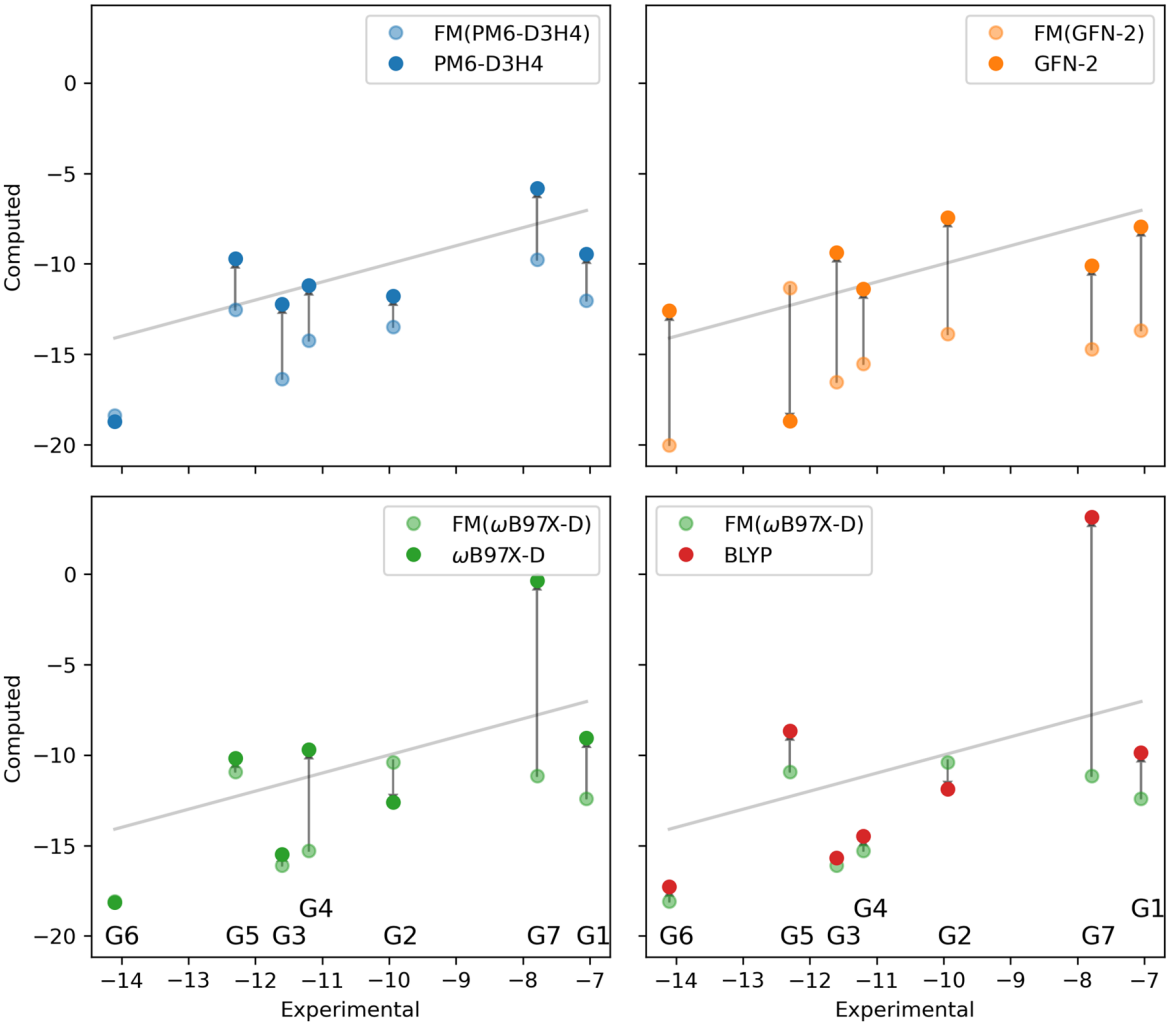
Plots of the experimental results vs. computed binding free energies before and after employing the QM/MM correction scheme. All energies are given in kcal/mol

Charge blurring, as a whole, worsened results. For BLYP/6-31G* calculations, there was a net increase in RMSD of about 0.5 kcal/mol, whereas for the $\omega$B97X-D/def2-SVP calculations the RMSD went up by about 2.1 kcal/mol after using blurring. These findings are not entirely unexpected, as results are often dependent on selecting the correct blur width, for which the correct choice is not always transferable among different QM levels of theory (e.g., the appropriate blur width for BLYP/6-31G* and $\omega$B97X-D/def2-SVP will not be the same) [68]. Overall, the SQM performance was markedly better than the DFT results. It is worth noting BLYP was included because of its good performance when computing solvation free energies in past works, but it is generally considered less robust in comparison to common methods (e.g., $\omega$B97X-D, B3LYP, etc.), and its poor results here are not too surprising [77]. The results for $\omega$B97X-D/def2-SVP, excluding G7 (RMSE $\approx$ 2.86 kcal/mol), show promise for this level of theory if executed correctly. A summary of pre- and post-QM/MM correction binding free energies can be found in Fig. 8 and plots illustrating the associated change in binding free energy for each guest can be found in Fig. 9.

### Metrics for the indirect approach

One of the most important steps in indirectly computing QM/MM free energy is to determine the degree to which results are converged. In particular, one way to check how well force-matching improved our indirect scheme is to compare how the metrics $\sigma _{\varDelta {U}}$ and $\varPi$ change when using a standard force field (e.g., CGenFF) versus a FM description. In principle, changing from CGenFF to FM should improve both statistics, with $\varPi$ getting large and $\sigma _{\varDelta {U}}$ getting smaller (i.e., $\varDelta \varPi >0$ and $\varDelta \sigma _{\varDelta {U}}<0$).

Since the force-matching is performed in gas-phase, one would expect improvements to be much larger in gas-phase than in solution. However, we anticipate that trends observed based on comparing the change in metrics when computing $\varDelta {A}_{\text {gas}}^{\text {MM}\rightarrow \text {QM}}$ should be consistent with $\varDelta {A}_{\text {aq}}^{\text {MM}\rightarrow \text {QM/MM}}$ (e.g., if the calculation fails when going from FM to QM in gas-phase, it will most likely fail for FM/MM to QM/MM in solution as well). The resulting metric changes for each guest in gas-phase is shown in Table 5 for PM6-D3H4 and GFN-2. Overall, the changes are somewhat modest, with guests G1, G2, G3, G4, and G6 demonstrating decent improvement. For guest G7, the quality of correction deteriorated when using a FM description, indicating a bad fit most likely stemming from overenthusiastic manual tweaking of dihedral multiplicities. This observation also explains why the DFT results were rather poor for G7.

The results for the four G5 compounds are rather perplexing. Although, in principle, the parameter sets between enantiomers with the same charge should be identical, the force-matching procedure produced subtle differences in the resulting fit for each enantiomer pair. This discrepancy is likely due to the inherent limitation of finite sampling. That is, the sampling of configurations between the two enantiomers is similar, but not perfectly identical due to different initial starting conditions (such as different random starting velocities). In the limit of infinite sampling, the ensembles would theoretically be identical. Coupling the initial deviations with the need to tweak dihedral multiplicities for each parameter set (e.g., the adjustments to multiplicities for *R*-G5 were different than the adjustments for *S*-G5), these factors only exacerbated the differences in the quality of FM fit. However, one must note that the quality of correction based on the metrics of Table 5 for the G5 derivatives seem to be Hamiltonian dependent, with GFN-2 based parameters only improving the correction as compared to the deterioration observed for the *R* enantiomers with the $\omega$B97X-D/def2-SVP based parameters. Since dihedral forms remain constant across Hamiltonians, this likely indicates that the fitting of dihedrals in the force-matching procedure will potentially have a large dependence on the Hamiltonian of interest (e.g., different levels of theory might require different dihedral multiplicities).

Small inconsistencies found within the ForceSolve software during the fitting procedure also attracted our attention, specifically regarding the need to remove electrostatic and van der Waals forces before the FM fitting. For neutral compounds, ForceSolve is able to remove all non-bonded forces internally. For charged compounds however, ForceSolve will arbitrarily neutralize the charge and therefore produce the wrong electrostatic forces. Thus, electrostatic forces should be removed prior to invoking ForceSolve, and then the force-matching should be performed with all partial charges set to 0. Whether the electrostatic forces are removed prior to invoking ForceSolve or handled by ForceSolve internally should not affect the fit. Unfortunately, it seems that intramolecular fitting results are better whenever ForceSolve is allowed to handle the electrostatic forces, as can be seen by the differences in residuals obtained for force-matching the CB[8] host (see SI, Table S4). Although the average differences is small ($\sim$ 0.3 kcal/mol$\cdot$Å), it will still contribute to less effective parameters for use in the indirect approach.

### Lessons learned

A few things become rather clear in retrospective. First, the level of QM/MM theory required to accurately compute binding free energies is not necessarily high. Rather, use of well-suited semi-empirical QM methods can perform fairly well at a fraction of the cost of DFT/ab-initio QM methods. Going forward, efforts towards employing SQM methods, such as PM6-D3H4 and GFN-2, and their furthering development into relevant software (e.g., including GFN-2 support into Psi4, and interfacing Psi4 with CHARMM) will be central to future challenges.

*Better methods for computing the QM/MM correction*, $\varDelta {A}^\mathbf{MM \rightarrow \mathbf{QM}/MM }$. In particular, faster and more accessible SQM Hamiltonians open the door to more sophisticated methods for computing free energy differences between different levels of theory. Specifically, use of non-equilibrium approaches for computing the free energy between MM and QM/MM has shown much promise. Methods such as fast-switching non-equilibrium work [78], in conjunction with the Jarzynski equality, will, in principle, guarantee the production of a converged free energy difference (so long as the switching lengths are long enough to bridge disparity between configurational spaces). By combining non-equilibrium approaches with force-matched potentials, the length of non-equilibrium switching simulations required to converge free energy differences between levels of theory can be drastically reduced [37].

*Force-Matching procedure can be improved* The poor overlap between MM and QM/MM is the biggest obstacle to the mainstream use of the indirect approach to QM/MM free energy. The use of force-matching seeks to address this problem by providing an improved “launching-point” for performing one-sided free energy calculations between levels of theory, but critical consideration is required to correctly ascertain FM parameters. The largest limitation to FM based approaches is the underlying sampling of the training set. Specifically, it is important to ask how representative of the target space the sampled configurations are. Although we have shown that our force-matching methods generally improved our FEP calculations, it stands to reason that classical gas-phase ensembles may be a poor training choice for improving overlap with QM/MM aqueous/bound state phase space. Thus, we seek to shift our force-matching to train on simulations of condensed phase systems in future work.

We also seek to explore the fitting of partial charges in future challenges. Potentially, charge assignment can be provided in a somewhat arbitrary, yet uniform manner (as was the case for the above force-matching procedure). Alternatively, partial charges could be assigned as a pre-step of the force-matching process, where charge populations are evaluated at the same time forces are computed on the relevant training set (e.g., taking the average of the partial charges on all snapshots). The fitting of partial charges in force-matching is a non-linear fit, which not only slows the procedure, but is unclear as to whether it can provide improvements for the use of FM in indirect QM/MM approaches [79].

And finally, as demonstrated, highly expensive QM/MM levels of theory are not necessarily required to produce excellent binding free energies. Using computationally less demanding SQM methods paves the way for using force-matching approaches on larger, more complex molecules of interest (e.g., generating FM potentials for both host and guest, and performing QM/MM corrections for host, guest, and host–guest complex).

*Better MM is needed to pair with better QM* An important concern regarding QM/MM approaches in binding free energies is how well the QM level of theory works with the classical force field. Often times, more sophisticated QM approaches are out-performed by simpler QM methods (e.g., semi-empirical QM, BLYP, etc.) due to beneficial “error cancellation” (e.g., TIP3 water tends to be over-polarized, whereas BLYP tends to underestimate polarization) [77]. This highlights the unfortunate truth that often, the classical MM description is the limiting factor for the accuracy of QM/MM calculations. With the goal of employing more sophisticated QM Hamiltonians in QM/MM descriptions, it is only natural to turn ourselves to polarizable molecular mechanics [80], which represent one more step to close the gap between MM and QM/MM.

## Conclusions

In this SAMPL challenge, we aimed to evaluate how well our indirect QM/MM free energy schemes performed on a blind dataset (particularly as QM/MM methods have historically not performed well in previous iterations of the SAMPL challenges). Our submissions showed how semi-empirical methods (GFN2, PM6-D3H4) can successfully provide accurate results at relatively low computational expense. The bridging capability of the indirect scheme coupled with force-matching procedure shone brightest at this intermediate level, allowing the correction scheme to reduce the RMSE of the binding free energies by more than 30%.

This work also sheds light on some of the more nuanced aspects of force-matching for indirect QM/MM free energies, and parameterization in general. In particular, adjusting torsional parameters in force-matched descriptions should be performed with great trepidation, as bad dihedral terms can still provide reasonable residuals at fit time, but skew overlaps with the targeted level of theory. It also illuminated the need for a critical evaluation of “best-practices” for force-matching with the goal of improving indirect QM/MM calculations in condensed phase systems.

And finally, the dominance of SQM Hamiltonians in our results highlights the feasibility of more sophisticated methods for computing free energy differences between levels of theory. Specifically, the ease and expedience with which modern SQM methods can be performed perfectly lends itself to the use of non-equilibrium methods for computing free energy between levels of theory. Pairing better one-sided approaches with enhancements to our force-matching methods will only yield improvement in the future.

## Supplementary Information

Below is the link to the electronic supplementary material.

Supplementary file1 (PDF 279 kb)

## Data Availability

Parameters, trajectories, and other relevant data-sets are available upon request to the corresponding author.

## Footnotes

1 QM herein is used to designate the use of either semi-empirical QM (SQM; e.g., PM6-D3H4 [47] or GFN-2 [48]) or ab-initio/density functional theory (DFT; e.g., BLYP [44, 49], $\omega$B97X-D [50], etc.) Hamiltonians.

2 The aforementioned workflow is effectively a procedure to computing the “unbinding free energy” (e.g., going from a bound guest to a guest free in solution)—which simply yields the opposite of the binding free energy.

3 The GFN-2 entry was omitted from the original verified submissions due to a minor scripting issue resolved (unfortunately) shortly after the submission deadline.

## References

[1] Jorgensen WL (2004). The many roles of computation in drug discovery. Science.

[2] Sliwoski G, Kothiwale S, Meiler J, Lowe EW (2014). Computational methods in drug discovery. Curr Opin Drug Discov Dev.

[3] Kollman P (1993). Free energy calculations: applications to chemical and biochemical phenomena. Chem Rev.

[4] Shirts MR (2012). Best practices in free energy calculations for drug design.

[5] Chipot C, Pohorille A (2007). Free energy calculations.

[6] Guthrie JP (2009). A blind challenge for computational solvation free energies: introduction and overview. J Phys Chem B.

[7] Geballe MT, Skillman AG, Nicholls A, Guthrie JP, Taylor PJ (2010). The SAMPL2 blind prediction challenge: introduction and overview. J Comput Aided Mol Des.

[8] Muddana HS, Varnado CD, Bielawski CW, Urbach AR, Isaacs L, Geballe MT, Gilson MK (2012). Blind prediction of host–guest binding affinities: a new SAMPL3 challenge. J Comput Aided Mol Des.

[9] Muddana HS, Fenley AT, Mobley DL, Gilson MK (2014). The SAMPL4 host–guest blind prediction challenge: an overview. J Comput Aided Mol Des.

[10] Yin J, Henriksen NM, Slochower DR, Shirts MR, Chiu MW, Mobley DL, Gilson MK (2017). Overview of the SAMPL5 host–guest challenge: are we doing better?. J Comput Aided Mol Des.

[11] Rizzi A, Murkli S, McNeill JN, Yao W, Sullivan M, Gilson MK, Chiu MW, Isaacs L, Gibb BC, Mobley DL, Chodera JD (2018). Overview of the SAMPL6 host–guest binding affinity prediction challenge. J Comput Aided Mol Des.

[12] Zacharias M, Straatsma TP, McCammon JA (1994). Separation-shifted scaling, a new scaling method for Lennard-Jones interactions in thermodynamic integration. J Chem Phys.

[13] Beutler TC, Mark AE, van Schaik RC, Gerber PR, van Gunsteren WF (1994). Avoiding singularities and numerical instabilities in free energy calculations based on molecular simulations. Chem Phys Lett.

[14] Gao J, Xia X (1992). A priori evaluation of aqueous polarization effects through Monte Carlo QM-MM simulations. Science.

[15] Gao J, Luque FJ, Orozco M (1993). Induced dipole moment and atomic charges based on average electrostatic potentials in aqueous solution. J Chem Phys.

[16] Luzhkov V, Warshel A (1992). Microscopic models for quantum mechanical calculations of chemical processes in solutions: LD/AMPAC and SCAAS/AMPAC calculations of solvation energies. J Comput Chem.

[17] Wesolowski T, Warshel A (1994). Ab initio free energy perturbation calculations of solvation free energy using the frozen density functional approach. J Phys Chem.

[18] Gao J, Freindorf M (1997). Hybrid ab initio QM/MM simulation of N-methylacetamide in aqueous solution. J Phys Chem A.

[19] Zheng YJ, Merz KM (1992). Mechanism of the human carbonic anhydrase II-catalyzed hydration of carbon dioxide. J Am Chem Soc.

[20] Zwanzig RW (1954). High-temperature equation of state by a perturbation method. I. Nonpolar gases. J Chem Phys.

[21] Liu S, Ruspic C, Mukhopadhyay P, Chakrabarti S, Zavalij PY, Isaacs L (2005). The cucurbit[n]uril family: prime components for self-sorting systems. J Am Chem Soc.

[22] Lagona J, Mukhopadhyay P, Chakrabarti S, Isaacs L (2005). The cucurbit[n]uril family. Angew Chem Int Ed.

[23] Mobley DM (2020) samplchallenges/SAMPL8: SAMPL8 CB8 “drugs of abuse” challenge inputs. 10.5281/zenodo.4029560. Last accessed 1 Sept 2021

[24] Mobley DL, Bergazin TD, Amezcua M (2021) The SAMPL8 blind prediction challenges for computational chemistry. https://github.com/samplchallenges/SAMPL8

[25] Csányi G, Albaret T, Payne MC, De Vita A (2004). Learn on the fly: a hybrid classical and quantum-mechanical molecular dynamics simulation. Phys Rev Lett.

[26] Akin-Ojo O, Song Y, Wang F (2008). Developing ab initio quality force fields from condensed phase quantum-mechanics/molecular-mechanics calculations through the adaptive force matching method. J Chem Phys.

[27] Akin-Ojo O, Wang F (2010). The quest for the best nonpolarizable water model from the adaptive force matching method. J Comput Chem.

[28] Wang LP, Van Voorhis T (2010). Communication: hybrid ensembles for improved force matching. J Chem Phys.

[29] Wang F, Akin-Ojo O, Pinnick E, Song Y (2011). Approaching post-Hartree–Fock quality potential energy surfaces with simple pair-wise expressions: parameterising point-charge-based force fields for liquid water using the adaptive force matching method. Mol Simul.

[30] Wang LP, Chen J, Van Voorhis T (2012). Systematic parametrization of polarizable force fields from quantum chemistry data. J Chem Theory Comput.

[31] Pinnick ER, Calderon CE, Rusnak AJ, Wang F (2012). Achieving fast convergence of ab initio free energy perturbation calculations with the adaptive force-matching method. Theor Chem Acc.

[32] Li J, Wang F (2015). Pairwise-additive force fields for selected aqueous monovalent ions from adaptive force matching. J Chem Phys.

[33] Wang LP, McKiernan KA, Gomes J, Beauchamp KA, Head-Gordon T, Rice JE, Swope WC, Martínez TJ, Pande VS (2017). Building a more predictive protein force field: a systematic and reproducible route to AMBER-FB15. J Phys Chem B.

[34] Brooks BR, Brooks CL, Mackerell AD, Nilsson L, Petrella RJ, Roux B, Won Y, Archontis G, Bartels C, Boresch S, Caflisch A, Caves L, Cui Q, Dinner AR, Feig M, Fischer S, Gao J, Hodoscek M, Im W, Kuczera K, Lazaridis T, Ma J, Ovchinnikov V, Paci E, Pastor RW, Post CB, Pu JZ, Schaefer M, Tidor B, Venable RM, Woodcock HL, Wu X, Yang W, York DM, Karplus M (2009). CHARMM: the biomolecular simulation program. J Comput Chem.

[35] Vanommeslaeghe K, Raman EP, MacKerell AD (2012). Automation of the CHARMM general force field (CGenFF) II: assignment of bonded parameters and partial atomic charges. J Chem Inf Model.

[36] Han K, Hudson PS, Jones MR, Nishikawa N, Tofoleanu F, Brooks BR (2018). Prediction of CB[8] host–guest binding free energies in SAMPL6 using the double-decoupling method. J Comput Aided Mol Des.

[37] Hudson PS, Boresch S, Rogers DM, Woodcock HL (2018). Accelerating QM/MM free energy computations via intramolecular force matching. J Chem Theory Comput.

[38] Deng CL, Murkli SL, Isaacs LD (2020). Supramolecular hosts as: in vivo sequestration agents for pharmaceuticals and toxins. Chem Soc Rev.

[39] ONeil M (2013). The Merck Index—an encyclopedia of chemicals, drugs, and biologicals.

[40] Vanommeslaeghe K, Hatcher E, Acharya C, Kundu S, Zhong S, Shim J, Darian E, Guvench O, Lopes P, Vorobyov I, Mackerell AD (2009). CHARMM general force field: a force field for drug-like molecules compatible with the CHARMM all-atom additive biological force fields. J Comput Chem.

[41] Møller C, Plesset MS (1934). Note on an approximation treatment for many-electron systems. Phys Rev.

[42] Francl MM, Pietro WJ, Hehre WJ, Binkley JS, Gordon MS, DeFrees DJ, Pople JA (1982). Self-consistent molecular orbital methods. xxiii. A polarization-type basis set for second-row elements. J Chem Phys.

[43] Becke AD (1993). Density-functional thermochemistry. III. The role of exact exchange. J Chem Phys.

[44] Lee C, Yang W, Parr RG (1988). Development of the Colle-Salvetti correlation-energy formula into a functional of the electron density. Phys Rev B.

[45] Marenich AV, Jerome SV, Cramer CJ, Truhlar DG (2012). Charge model 5: an extension of Hirshfeld population analysis for the accurate description of molecular interactions in gaseous and condensed phases. J Chem Theory Comput.

[46] Miertuš S, Scrocco E, Tomasi J (1981). Electrostatic interaction of a solute with a continuum. A direct utilization of AB initio molecular potentials for the prevision of solvent effects. Chem Phys.

[47] Řezáč J, Hobza P (2012). Advanced corrections of hydrogen bonding and dispersion for semiempirical quantum mechanical methods. J Chem Theory Comput.

[48] Bannwarth C, Ehlert S, Grimme S (2019). GFN2-xTB–an accurate and broadly parametrized self-consistent tight-binding quantum chemical method with multipole electrostatics and density-dependent dispersion contributions. J Chem Theory Comput.

[49] Becke AD (1988). Density-functional exchange-energy approximation with correct asymptotic behavior. Phys Rev A.

[50] Chai JD, Head-Gordon M (2008). Long-range corrected hybrid density functionals with damped atom–atom dispersion corrections. Phys Chem Chem Phys.

[51] Smith DG, Burns LA, Simmonett AC, Parrish RM, Schieber MC, Galvelis R, Kraus P, Kruse H, Di Remigio R, Alenaizan A, James AM, Lehtola S, Misiewicz JP, Scheurer M, Shaw RA, Schriber JB, Xie Y, Glick ZL, Sirianni DA, OBrien JS, Waldrop JM, Kumar A, Hohenstein EG, Pritchard BP, Brooks BR, Schaefer HF, Sokolov AY, Patkowski K, Deprince AE, Bozkaya U, King RA, Evangelista FA, Turney JM, Crawford TD, Sherrill CD (2020). P SI4 1.4: open-source software for high-throughput quantum chemistry. J Chem Phys.

[52] Stewart JJP (2016). MOPAC2016.

[53] Rogers DM (2016) ForceSolve. https://github.com/frobnitzem/forcesolve

[54] Hudson PS, Han K, Woodcock HL, Brooks BR (2018). Force matching as a stepping stone to QM/MM CB[8] host/guest binding free energies: a SAMPL6 cautionary tale. J Comput Aided Mol Des.

[55] Shin WH, Kim J, Kim D, Seok C (2013). GalaxyDock2: protein-ligand docking using beta-complex and global optimization. J Comput Chem.

[56] Baek M, Shin WH, Chung HW, Seok C (2017). GalaxyDock BP2 Score: a hybrid scoring function for accurate protein-ligand docking. J Comput Aided Mol Design.

[57] Eastman P, Swails J, Chodera JD, McGibbon RT, Zhao Y, Beauchamp KA, Wang LP, Simmonett AC, Harrigan MP, Stern CD, Wiewiora RP, Brooks BR, Pande VS (2017). OpenMM 7: rapid development of high performance algorithms for molecular dynamics. PLoS Comput Biol.

[58] Gilson M, Given J, Bush B, McCammon J (1997). The statistical-thermodynamic basis for computation of binding affinities: a critical review. Biophys J.

[59] Boresch S, Tettinger F, Leitgeb M, Karplus M (2003) Absolute binding free energies: a quantitative approach for their calculation. J Phys Chem B 107(35):9535–9551

[60] Krämer A, Hudson PS, Jones MR, Brooks BR (2020). Multi-phase Boltzmann weighting: accounting for local inhomogeneity in molecular simulations of water-octanol partition coefficients in the SAMPL6 challenge. J Comput Aided Mol Des.

[61] Krämer A, Ghysels A, Wang E, Venable RM, Klauda JB, Brooks BR, Pastor RW (2020). Membrane permeability of small molecules from unbiased molecular dynamics simulations. J Chem Phys.

[62] Shirts MR, Chodera JD (2008). Statistically optimal analysis of samples from multiple equilibrium states. J Chem Phys.

[63] Hermans J, Wang L (1997). Inclusion of loss of translational and rotational freedom in theoretical estimates of free energies of binding. Application to a complex of benzene and mutant T4 lysozyme. J Am Chem Soc.

[64] Zhang Y, McCammon JA (2003). Studying the affinity and kinetics of molecular association with molecular-dynamics simulation. J Chem Phys.

[65] Han K, Hudson PS, Jones MR, Nishikawa N, Tofoleanu F, Brooks BR (2018). Prediction of CB[8] host–guest binding free energies in SAMPL6 using the double-decoupling method. J Comput Aided Mol Des.

[66] Lee J, Miller BT, Brooks BR (2016). Computational scheme for pH-dependent binding free energy calculation with explicit solvent. Protein Sci.

[67] Lee J, Tofoleanu F, Pickard FC, König G, Huang J, Damjanović A, Baek M, Seok C, Brooks BR (2017). Absolute binding free energy calculations of CBClip host–guest systems in the SAMPL5 blind challenge. J Comput Aided Mol Des.

[68] Das D, Eurenius KP, Billings EM, Sherwood P, Chatfield DC, Hodošček M, Brooks BR (2002). Optimization of quantum mechanical molecular mechanical partitioning schemes: Gaussian delocalization of molecular mechanical charges and the double link atom method. J Chem Phys.

[69] Epifanovsky E, Gilbert AT, Feng X, Lee J, Mao Y, Mardirossian N, Pokhilko P, White AF, Coons MP, Dempwolff AL (2021). Software for the frontiers of quantum chemistry: an overview of developments in the Q-Chem 5 package. J Chem Phys.

[70] König G, Hudson PS, Boresch S, Woodcock HL (2014). Multiscale free energy simulations: an efficient method for connecting classical MD simulations to QM or QM/MM free energies using non-Boltzmann Bennett reweighting schemes. J Chem Theory Comput.

[71] Wu D, Kofke DA (2004). Model for small-sample bias of free-energy calculations applied to Gaussian-distributed nonequilibrium work measurements. J Chem Phys.

[72] Wu D, Kofke DA (2005). Phase-space overlap measures. I. Fail-safe bias detection in free energies calculated by molecular simulation. J Chem Phys.

[73] Boresch S, Woodcock HL (2016). Convergence of single-step free energy perturbation. Mol Phys.

[74] Caldararu O, Olsson MA, Wang M, Ryde U (2018). Binding free energies in the SAMPL6 octa-acid host-guest challenge calculated with MM and QM methods. J Comput Aided Mol Des.

[75] Stigler SM (1989). Francis Galtons account of the invention of correlation. Stat Sci.

[76] Kendall MG (1938). A new measure of rank correlation. Biometrika.

[77] König G, Mei Y, Pickard FC, Simmonett AC, Miller BT, Herbert JM, Woodcock HL, Brooks BR, Shao Y (2016). Computation of hydration free energies using the multiple environment single system quantum mechanical/molecular mechanical method. J Chem Theory Comput.

[78] Hudson PS, Woodcock HL, Boresch S (2015). Use of nonequilibrium work methods to compute free energy differences between molecular mechanical and quantum mechanical representations of molecular systems. J Phys Chem Lett.

[79] Giese TJ, York DM (2019). Development of a robust indirect approach for MM→ QM free energy calculations that combines force-matched reference potential and Bennetts acceptance ratio methods. J Chem Theory Comput.

[80] Ponder JW, Wu C, Ren P, Pande VS, Chodera JD, Schnieders MJ, Haque I, Mobley DL, Lambrecht DS, DiStasio RA, Head-Gordon M, Clark GNI, Johnson ME, Head-Gordon T (2010). Current status of the amoeba polarizable force field. J Phys Chem B.

