# Supplementary material for: Obtaining QM/MM binding free energies in the SAMPL8 drugs of abuse challenge: indirect approaches
Source: J Comput Aided Mol Des. 2022 May 22;36(4):263–77. doi: 10.1007/s10822-022-00443-8 (PMC9148874; doi:10.1007/s10822-022-00443-8)
Supplement: Supplementary file 1 — Supplementary file1 (PDF 279 kb) [file 10822_2022_443_MOESM1_ESM.pdf]

Noname manuscript No.
(will be inserted by the editor)

Obtaining QM/MM Binding Free Energies in the SAMPL8 Drugs of Abuse Challenge: Indirect Approaches- Supplmental Information

Phillip S. Hudson[1] · Felix Aviat[1] · Rubén Meana-Pañeda[1] · Luke Warrensford[2] · Benjamin C. Pollard[2] · Samarjeet Prasad[1] · Michael R. Jones[1] · H. Lee Woodock[2] · Bernard R. Brooks[1]

Received: date / Accepted: date

Phillip S. Hudson
E-mail: phillip.hudson@nih.gov

1

Laboratory of Computational Biology, National Heart, Lung and Blood Institute, National Institutes of Health, Bethesda, MD 20852, USA

2

Department of Chemistry, University of South Florida, Tampa, Florida 33620, USA

Table S1 Summary of literature values for pK_as of the guest compounds

Molecule	Designation	pK_a (tabulated)	Ref.
Methamphetamine	G1	9.9 (25C)	[1]
Fentanyl	G2	9.0	[2]
Morphine	G3	8.1	[2]
Hydromorphone	G4	8.6	[3]
Ketamine	G5	7.5	[4]
Phencyclidine	G6	8.3	[5]
Cocaine	G7	8.6 (15C)	[6]

S1 Charge Benchmark

In order to best reproduce the partial charges from the CHARMM Generalized Force Field (CGenFF[7]), partial charges of 19 small rigid molecules (fig. S1) found in CGenFF were computed using various methods, basis sets, solvent models and charge schemes. IQMol was used to generate structures, and QM single point energies / charge population anaylsis was performed with Q-Chem[8]. Partial charge RMSD of the QM calculations with respect to CGenFF was computed with (S2) and without hydrogens (S3). The partial charge RMSD was larger for "heavy-atoms only" as compared to the RMSD with all atoms. The HF method coupled with PCM implicit solvation and CM5-S charge scheme provided the lowest RMSD. Basis set 6-311G** was chosen due to excellent performance and better coverage over a wide variety of moieties.

Table S2 RMSD with respect to the CgenFF partial charges computed for the heavy atoms only, sorted by method, basis set, solvent model and charge scheme.

Method	Basis Set	Solvent	Charge Type	RMSD
B3LYP[9, 10]	6-311G**[11, 12]	NONE	CM5-S[13]	0.09
			RESP[14]	0.23
		PCM[15]	CM5-S	0.08
			RESP	0.23
	6-31G*	NONE	CM5-S	0.08
			RESP	0.26
		PCM	CM5-S	0.08
			RESP	0.21
		SM8[16]	CM5-S	0.08
			RESP	0.21
BLYP[17, 18]	6-311G**	NONE	CM5-S	0.09
			RESP	0.22
		PCM	CM5-S	0.08
			RESP	0.22
	6-31G*	NONE	CM5-S	0.09
			RESP	0.25
		PCM	CM5-S	0.08
			RESP	0.20
		SM8	CM5-S	0.08
			RESP	0.20
CAM-B3LYP[19]	6-311G**	NONE	CM5-S	0.09
			RESP	0.23
		PCM	CM5-S	0.08
			RESP	0.24
	6-31G*	NONE	CM5-S	0.08
			RESP	0.27
		PCM	CM5-S	0.08
			RESP	0.22
		SM8	CM5-S	0.08
			RESP	0.21
HF[20]	6-311G**	NONE	CM5-S	0.08
			RESP	0.25
		PCM	CM5-S	0.08
			RESP	0.26
	6-31G*	NONE	CM5-S	0.08
			RESP	0.30
		PCM	CM5-S	0.07
			RESP	0.25
		SM8	CM5-S	0.08
			RESP	0.24
M06-2X[21]	6-311G**	NONE	CM5-S	0.08
			RESP	0.24
		PCM	CM5-S	0.08
			RESP	0.25
	6-31G*	NONE	CM5-S	0.08
			RESP	0.28
		PCM	CM5-S	0.08
			RESP	0.22
		SM8	CM5-S	0.08
			RESP	0.22
ωB97X-D[22]	6-311G**	NONE	CM5-S	0.09
			RESP	0.24
		PCM	CM5-S	0.08
			RESP	0.24
	6-31G*	NONE	CM5-S	0.08
			RESP	0.22
		PCM	CM5-S	0.08
			RESP	0.23
		SM8	CM5-S	0.08
			RESP	0.23

Table S3 RMSD with respect to the CgenFF partial charges computed for all atoms (including hydrogens), sorted by method, basis set, solvent model and charge scheme.

Method	Basis Set	Solvent	Charge Type	RMSD
B3LYP[9, 10]	6-311G**[11, 12]	NONE	CM5-S[13]	0.06
			RESP[14]	0.15
		PCM[15]	CM5-S	0.05
			RESP	0.16
	6-31G*	NONE	CM5-S	0.06
			RESP	0.14
		PCM	CM5-S	0.05
			RESP	0.14
		SM8	CM5-S	0.05
			RESP	0.14
BLYP[17, 18]	6-311G**	NONE	CM5-S	0.06
			RESP	0.14
		PCM	CM5-S	0.06
			RESP	0.15
	6-31G*	NONE	CM5-S	0.06
			RESP	0.13
		PCM	CM5-S	0.06
			RESP	0.13
		SM8	CM5-S	0.06
			RESP	0.13
CAM-B3LYP[19]	6-311G**	NONE	CM5-S	0.06
			RESP	0.16
		PCM	CM5-S	0.05
			RESP	0.16
	6-31G*	NONE	CM5-S	0.06
			RESP	0.14
		PCM	CM5-S	0.05
			RESP	0.15
		SM8	CM5-S	0.05
			RESP	0.14
HF[20]	6-311G**	NONE	CM5-S	0.06
			RESP	0.17
		PCM	CM5-S	0.05
			RESP	0.18
	6-31G*	NONE	CM5-S	0.05
			RESP	0.16
		PCM	CM5-S	0.05
			RESP	0.17
		SM8	CM5-S	0.05
			RESP	0.16
M06-2X[21]	6-311G**	NONE	CM5-S	0.06
			RESP	0.16
		PCM	CM5-S	0.05
			RESP	0.17
	6-31G*	NONE	CM5-S	0.06
			RESP	0.15
		PCM	CM5-S	0.05
			RESP	0.15
		SM8	CM5-S	0.05
			RESP	0.15
ωB97X-D[22]	6-311G**	NONE	CM5-S	0.06
			RESP	0.16
		PCM	CM5-S	0.05
			RESP	0.16
	6-31G*	NONE	CM5-S	0.06
			RESP	0.15
		PCM	CM5-S	0.05
			RESP	0.15
		SM8	CM5-S	0.05
			RESP	0.15

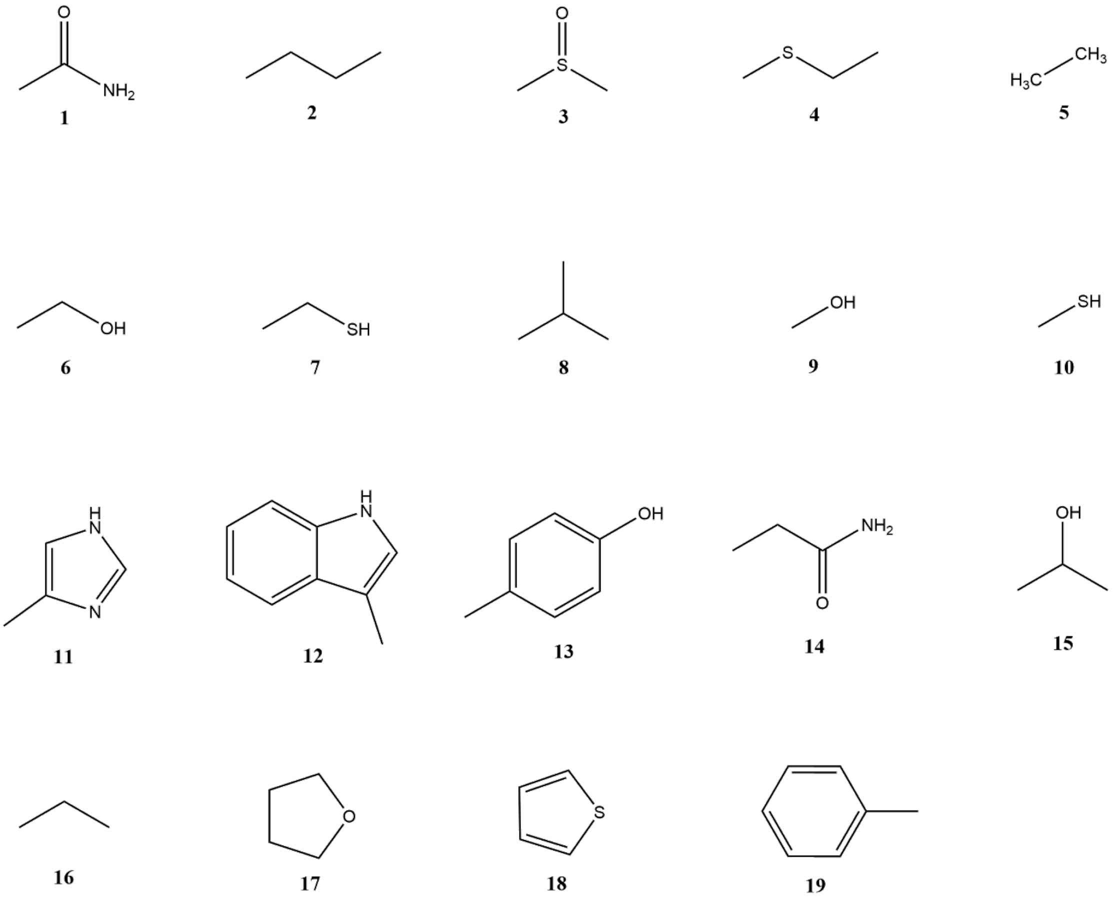

Fig. S1 Molecules found in CGenFF used for the partial charge benchmark.

Table S4 Table of per-atom residuals for Cucurbit-[8]-ural with electrostatic forces removed by either ForceSolve or preprocessed. All residuals are in units of kcal/mol·Å

Atom Type	$\hat{F}_{ELEC}$ removed in ForceSolve	$\hat{F}_{ELEC}$ removed prior to ForceSolve
HGA1CB	3.01	3.04
CG2RCB	2.46	2.90
NG2RCB	2.90	2.98
CG3RCB	2.19	2.31
OG2DCB	1.51	2.20
HGA2CB	5.70	6.13
CG32CB	2.37	2.47
Average	2.87±1.34	3.15±1.36

Table S5 Distribution of the r_{max} values for all guests and all force-matched force fields used. $\%_{>}$ designates the percentage of snapshots for which the distance between the guest and the host center of masses was above 5.5 Å. The free energy contributions $\Delta A_{\lambda=0}^{\mathrm{restr.\ off}}$ are in kcal/mol. For the ketamine molecule (guest G5), letters 'R' and 'S' designate the R and S enantiomers, and superscripts $\circ$ and $+$ designate the neutral and protonated molecule, respectively.

Force field	FM(GFN2)				FM(PM6)				FM(ωB97XD)			
Guest	r_{max}	r_{min}	$\%_{>}$	$\Delta A_{\lambda=0}^{\mathrm{restr.\ off}}$	r_{max}	r_{min}	$\%_{>}$	$\Delta A_{\lambda=0}^{\mathrm{restr.\ off}}$	r_{max}	r_{min}	$\%_{>}$	$\Delta A_{\lambda=0}^{\mathrm{restr.\ off}}$
G1	3.21	0.20	0	0	2.67	0.12	0	0	3.43	0.15	0	0
G2	6.97	0.23	30.5	-0.18 ± 0.16	7.16	0.21	36.2	-0.27 ± 0.24	6.77	0.07	28.9	-0.09 ± 0.06
G3	2.15	0.15	0	0	1.84	0.16	0	0	2.04	0.22	0	0
G4	1.74	0.25	0	0	7.23	0.27	3.7	-0.18 ± 0.22	2.09	0.23	0	0
R-G5°	2.97	0.27	0	0	3.49	0.15	0	0	2.21	0.14	0	0
S-G5°	3.05	0.18	0	0	3.33	0.11	0	0	3.18	0.18	0	0
R-G5^{+}	2.43	0.18	0	0	3.65	0.16	0	0	3.35	0.17	0	0
S-G5^{+}	2.83	0.19	0	0	3.54	0.13	0	0	5.97	0.18	0.04	0.0 ± 0.0
G6	5.61	0.11	0.01	0.0 +/- 0.0	6.55	0.24	0.12	0.0 ± 0.0	4.96	0.25	0	0
G7	5.10	0.22	0	0	5.84	0.12	0.04	0.0 ± 0.0	3.88	0.22	0	0

References

1. Perrin DD (1965) Dissociation Constants of Organic Bases in Aqueous Solution. Butterworths
2. Roy SD, Flynn GL (1989) Solubility behavior of narcotic analgesics in aqueous media: solubilities and dissociation constants of morphine, fentanyl, and sufentanil. Pharmaceutical research 6(2):147–151, DOI 10.1023/A:1015932610010
3. Dioumaeva I, Hughes JM (2012) SAMHSA-Compliant LC/MS/MS Analysis of Opiates (Morphine and Codeine) in Urine with Agilent Bond ElutPlexa PCX and Agilent Poroshell 120. Agilent Technologies, Inc., URL https://www.agilent.com/cs/library/applications/5990-9625EN.pdf, Publication number 5990-9625EN
4. O'Neil Me (2013) The Merck Index - An Encyclopedia of Chemicals, Drugs, and Biologicals, Cambridge, UK: Royal Society of Chemistry, p 982
5. Sangster J (1994) Octanol-Water Partition Coefficients: Fundamentals and Physical Chemistry. Wiley, New York
6. (1996) The Merck Index, 12th edn. NewYork: Merck, ISBN0911910-12-3
7. Vanommeslaeghe K, Hatcher E, Acharya C, Kundu S, Zhong S, Shim J, Darian E, Guvench O, Lopes P, Vorobyov I, Mackerell AD (2009) CHARMM general force field: A force field for drug-like molecules compatible with the CHARMM all-atom additive biological force fields. J Comput Chem 31(4):NA–NA, DOI 10.1002/jcc.21367, URL https://doi.org/10.1002/jcc.21367
8. Epifanovsky E, Gilbert AT, Feng X, Lee J, Mao Y, Mardirossian N, Pokhilko P, White AF, Coons MP, Dempwolff AL, et al (2021) Software for the frontiers of quantum chemistry: An overview of developments in the q-chem 5 package. J Chem Phys 155(8):084801

9. Becke AD (1993) Density-functional thermochemistry. III. the role of exact exchange. J Chem Phys 98(7):5648–5652, DOI 10.1063/1.464913, URL https://doi.org/10.1063/1.464913
10. Lee C, Yang W, Parr RG (1988) Development of the colle-salvetti correlation-energy formula into a functional of the electron density. Phys Rev B 37(2):785–789, DOI 10.1103/physrevb.37.785, URL https://doi.org/10.1103/physrevb.37.785
11. Hariharan PC, Pople JA (1973) The influence of polarization functions on molecular orbital hydrogenation energies. Theor Chimica Acta 28(3):213–222, DOI 10.1007/bf00533485, URL https://doi.org/10.1007/bf00533485
12. Francl MM, Pietro WJ, Hehre WJ, Binkley JS, Gordon MS, DeFrees DJ, Pople JA (1982) Self-consistent molecular orbital methods. XXIII. a polarization-type basis set for second-row elements. J Chem Phys 77(7):3654–3665, DOI 10.1063/1.444267, URL https://doi.org/10.1063/1.444267
13. Marenich AV, Jerome SV, Cramer CJ, Truhlar DG (2012) Charge model 5: An extension of hirshfeld population analysis for the accurate description of molecular interactions in gaseous and condensed phases. Journal of Chemical Theory and Computation 8(2):527–541, DOI 10.1021/ct200866d
14. Bayly CI, Cieplak P, Cornell W, Kollman PA (1993) A well-behaved electrostatic potential based method using charge restraints for deriving atomic charges: The RESP model. J Phys Chem 97(40):10269–10280, DOI 10.1021/j100142a004, URL https://doi.org/10.1021/j100142a004
15. Miertuš S, Scrocco E, Tomasi J (1981) Electrostatic interaction of a solute with a continuum. A direct utilizaion of AB initio molecular potentials for the prevision of solvent effects. Chemical Physics 55(1):117–129, DOI 10.1016/0301-0104(81)85090-2
16. Marenich AV, Olson RM, Kelly CP, Cramer CJ, Truhlar DG (2007) Self-consistent reaction field model for aqueous and nonaqueous solutions based on accurate polarized partial charges.

Journal of Chemical Theory and Computation 3(6):2011–2033, DOI 10.1021/ct7001418

17. Becke AD (1988) Density-functional exchange-energy approximation with correct asymptotic behavior. Phys Rev A 38:3098–3100, DOI 10.1103/PhysRevA.38.3098, URL https://link.aps.org/doi/10.1103/PhysRevA.38.3098
18. Lee C, Yang W, Parr RG (1988) Development of the colle-salvetti correlation-energy formula into a functional of the electron density. Phys Rev B 37:785–789, DOI 10.1103/PhysRevB.37.785, URL https://link.aps.org/doi/10.1103/PhysRevB.37.785
19. Yanai T, Tew DP, Handy NC (2004) A new hybrid exchange-correlation functional using the Coulomb-attenuating method (CAM-B3LYP). Chemical Physics Letters 393(1-3):51–57, DOI 10.1016/j.cplett.2004.06.011
20. Artree DRH, Artree (WH (1935) Self-consistent field, with exchange, for beryllium. Proceedings of the Royal Society of London Series A - Mathematical and Physical Sciences 150(869):9–33, DOI 10.1098/rspa.1935.0085, URL https://royalsocietypublishing.org/
21. Zhao Y, Truhlar DG (2008) The M06 suite of density functionals for main group thermochemistry, thermochemical kinetics, noncovalent interactions, excited states, and transition elements: Two new functionals and systematic testing of four M06-class functionals and 12 other function. Theoretical Chemistry Accounts 120(1-3):215–241, DOI 10.1007/s00214-007-0310-x
22. Chai JD, Head-Gordon M (2008) Long-range corrected hybrid density functionals with damped atom-atom dispersion corrections. Physical Chemistry Chemical Physics 10(44):6615–6620, DOI 10.1039/b810189b
